# Supplementary material for: Change in Physical Activity after Diagnosis of Diabetes or Hypertension: Results from an Observational Population-Based Cohort Study
Source: Int J Environ Res Public Health. 2019 Nov 1;16(21):4247. doi: 10.3390/ijerph16214247 (PMC6862551; doi:10.3390/ijerph16214247)
Supplement: Supplementary file 1 [file ijerph-16-04247-s001.pdf]

International Journal of *Environmental Research and Public Health*

MDPI

Supplement

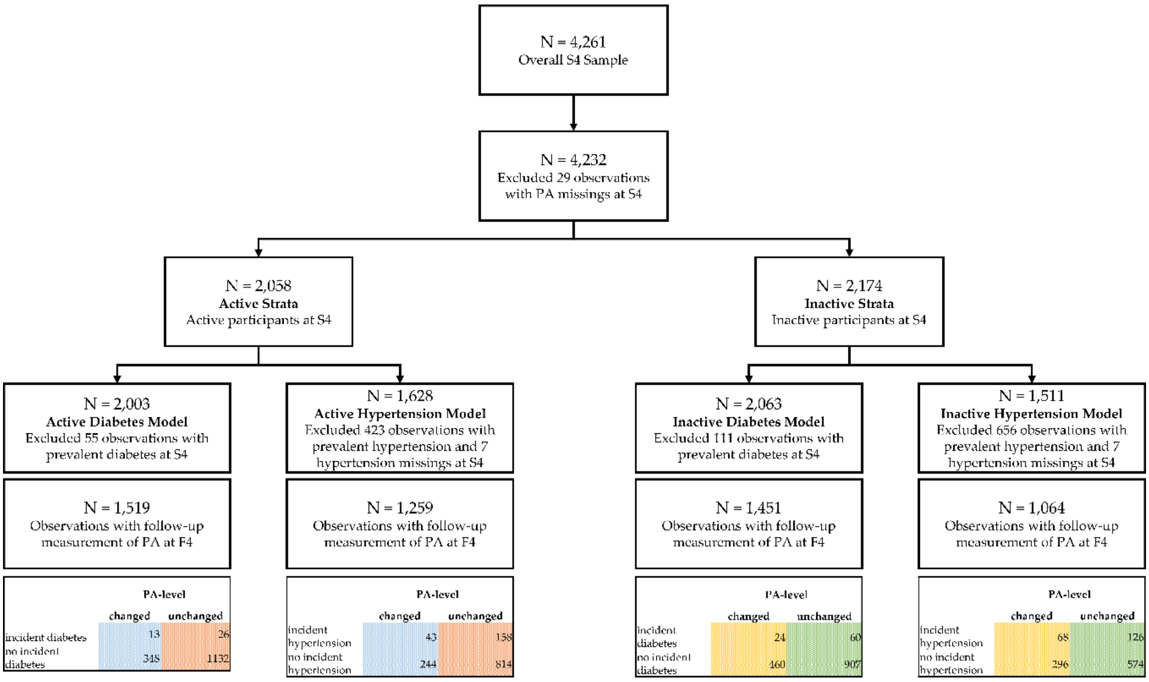

Figure S1. Add-on for Figure 2. Description of sample size for the first follow-up period from S4 to F4. Annotations: N = number of participants, PA = physical activity, S4 = baseline study (2000); F4 = first follow-up (2007); FF4 = second follow-up (2014).

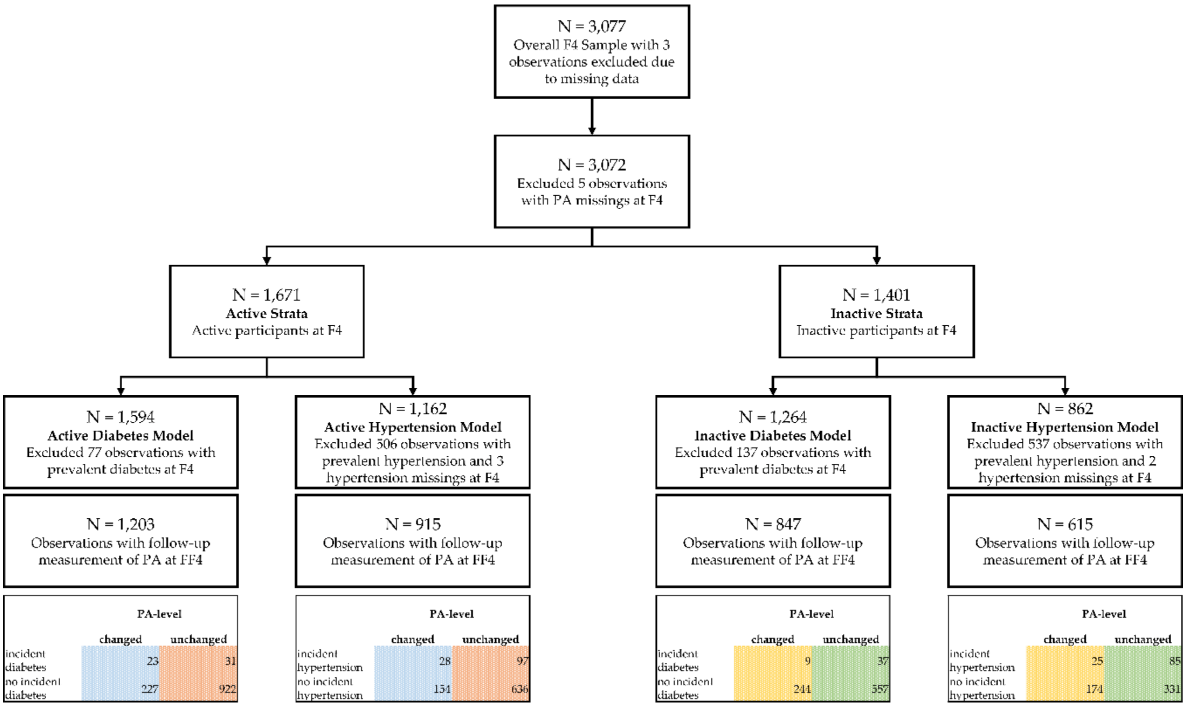

Figure S2. Add-on for Figure 2. Description of sample size for the second follow-up period from F4 to FF4. Annotations: N = number of participants, PA = physical activity, S4 = baseline study (2000); F4 = first follow-up (2007); FF4 = second follow-up (2014).

Table S1. In addition to Figure 3. Complete summary of the four logistic regression models with all covariates.

		95%CI	
	**OR**	**LL**	**UL**
Model: active, diabetes			
(Intercept)	0.309	0.222	0.429
hs.diab	2.161	1.202	3.888
sexF	1.086	0.858	1.374
scale(age)	0.903	0.793	1.030
fam.together	0.723	0.551	0.950
schulintermediate	0.837	0.629	1.114
schulhigher	0.699	0.517	0.944
scale(bmi)	1.325	1.168	1.504
scale(bmi.diff)	1.218	1.090	1.361
scale(pqol.diff)	0.940	0.840	1.051
scale(mqol.diff)	0.916	0.818	1.024
Model: active, hypertension			
(Intercept)	0.272	0.185	0.401
hs.hyp	0.929	0.639	1.350
sexF	1.074	0.822	1.402
scale(age)	0.830	0.714	0.965
fam.together	0.729	0.536	0.991
schulintermediate	1.014	0.733	1.402
schulhigher	0.779	0.558	1.089
scale(bmi)	1.306	1.131	1.508
scale(bmi.diff)	1.215	1.073	1.377
scale(pqol.diff)	0.944	0.830	1.074
scale(mqol.diff)	0.922	0.811	1.048
Model: inactive, diabetes			
(Intercept)	0.278	0.211	0.366
hs.diab	0.738	0.460	1.184
sexF	1.279	1.045	1.564
scale(age)	0.933	0.832	1.046
fam.together	1.494	1.169	1.910
schulintermediate	1.255	0.978	1.610
schulhigher	1.202	0.932	1.552
scale(bmi)	0.887	0.795	0.989
scale(bmi.diff)	0.870	0.784	0.965
scale(pqol.diff)	1.069	0.965	1.184
scale(mqol.diff)	1.130	1.021	1.250
Model: inactive, diabetes			
(Intercept)	0.267	0.193	0.370
hs.hyp	1.023	0.747	1.400
sexF	1.428	1.130	1.803
scale(age)	0.984	0.859	1.126
fam.together	1.457	1.100	1.930
schulintermediate	1.449	1.089	1.927
schulhigher	1.213	0.906	1.623

scale(bmi)	0.916	0.810	1.036
scale(bmi.diff)	0.883	0.786	0.992
scale(pqol.diff)	1.072	0.952	1.207
scale(mqol.diff)	1.187	1.054	1.336

Annotations: OR = odds ratio, 95%CI = 95% confidence interval, LL = lower limit, UL = upper limit

Note that all scaled variables have been standardized with mean equal to zero and standard deviation equal to one.

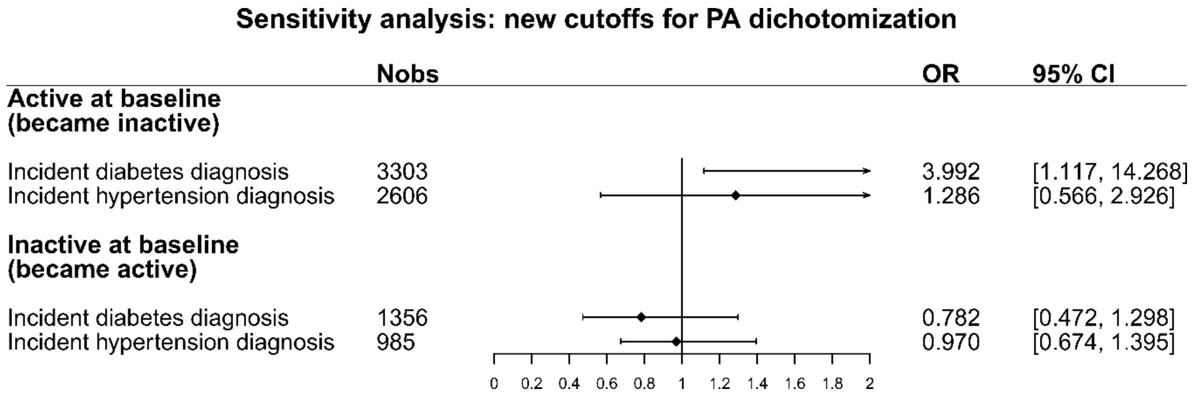

Figure S3. Sensitivity analysis. This figure is based on new defined cutoff points for the PA dichotomization. The odds for changing PA after a diabetes/hypertension diagnosis for both baseline PA strata. OR's for the active stratum display the odds of changing to inactive. OR's for the inactive stratum display the odds of changing to active.

Table S2. Sensitivity Analysis: Results of the models only including the first follow-up period from S4 to F4.

		95%CI	
	**OR**	**LL**	**UL**
Model: active, diabetes			
(Intercept)	0.216	0.083	0.557
hs.hyp	1.497	0.700	3.043
sexF	1.136	0.886	1.458
scale(age)	0.987	0.976	0.997
fam.together	0.774	0.583	1.032
schulintermediate	0.894	0.658	1.209
schulhigher	0.742	0.536	1.023
scale(bmi)	1.045	1.013	1.077
scale(bmi.diff)	1.095	1.023	1.172
scale(pqol.diff)	0.993	0.978	1.007
scale(mqol.diff)	0.985	0.973	0.997
Model: active, hypertension			
(Intercept)	0.266	0.088	0.804
hs.hyp	0.938	0.616	1.406
sexF	1.157	0.876	1.532
scale(age)	0.980	0.967	0.992
fam.together	0.776	0.568	1.068
schulintermediate	1.091	0.777	1.528

schulhigher	0.854	0.597	1.216
scale(bmi)	1.043	1.005	1.082
scale(bmi.diff)	1.102	1.022	1.189
scale(pqol.diff)	0.993	0.976	1.010
scale(mqol.diff)	0.986	0.972	1.000
Model: inactive, diabetes			
(Intercept)	0.511	0.213	1.222
hs.hyp	0.781	0.443	1.327
sexF	1.264	1.001	1.598
scale(age)	0.997	0.988	1.006
fam.together	1.545	1.165	2.064
schulintermediate	1.269	0.951	1.690
schulhigher	1.018	0.746	1.382
scale(bmi)	0.987	0.962	1.013
scale(bmi.diff)	0.956	0.904	1.011
scale(pqol.diff)	1.011	0.998	1.025
scale(mqol.diff)	1.011	1.000	1.023
Model: inactive, hypertension			
(Intercept)	0.391	0.139	1.096
hs.hyp	1.266	0.876	1.820
sexF	1.339	1.018	1.762
scale(age)	0.999	0.988	1.011
fam.together	1.462	1.052	2.050
schulintermediate	1.392	0.997	1.940
schulhigher	1.037	0.722	1.481
scale(bmi)	0.990	0.959	1.022
scale(bmi.diff)	0.967	0.906	1.033
scale(pqol.diff)	1.018	1.002	1.034
scale(mqol.diff)	1.018	1.005	1.032

Annotations: OR = odds ratio, 95%CI = 95% confidence interval, LL = lower limit, UL = upper limit

Note that all scaled variables have been standardized with mean equal to zero and standard deviation equal to one.

Table S3. Sensitivity Analysis: Results of the models only including the second follow-up period from F4 to FF4.

		95%CI	
	**OR**	**LL**	**UL**
Model: active, diabetes			
(Intercept)	0.030	0.008	0.104
hs.hyp	2.168	1.133	4.098
sexF	0.968	0.710	1.318
scale(age)	1.011	0.998	1.026
fam.together	0.795	0.554	1.152
schulintermediate	0.802	0.551	1.159
schulhigher	0.707	0.476	1.041
scale(bmi)	1.068	1.031	1.106

scale(bmi.diff)	1.140	1.043	1.248
scale(pqol.diff)	0.992	0.973	1.011
scale(mqol.diff)	1.003	0.986	1.019
Model: active, hypertension			
(Intercept)	0.030	0.006	0.135
hs.hyp	0.978	0.581	1.600
sexF	0.913	0.637	1.309
scale(age)	1.009	0.992	1.027
fam.together	0.798	0.524	1.237
schulintermediate	0.914	0.587	1.412
schulhigher	0.731	0.465	1.138
scale(bmi)	1.073	1.026	1.123
scale(bmi.diff)	1.129	1.019	1.251
scale(pqol.diff)	0.991	0.969	1.014
scale(mqol.diff)	1.005	0.985	1.025
Model: inactive, diabetes			
(Intercept)	1.395	0.392	4.985
hs.hyp	0.676	0.278	1.477
sexF	1.219	0.884	1.683
scale(age)	0.992	0.979	1.005
fam.together	1.302	0.884	1.941
schulintermediate	1.241	0.825	1.859
schulhigher	1.539	1.041	2.272
scale(bmi)	0.961	0.927	0.995
scale(bmi.diff)	0.904	0.828	0.984
scale(pqol.diff)	1.000	0.981	1.020
scale(mqol.diff)	1.013	0.995	1.031
Model: inactive, hypertension			
(Intercept)	0.711	0.153	3.289
hs.hyp	0.684	0.386	1.181
sexF	1.507	1.038	2.195
scale(age)	0.996	0.978	1.013
fam.together	1.433	0.916	2.278
schulintermediate	1.484	0.930	2.363
schulhigher	1.496	0.952	2.348
scale(bmi)	0.973	0.931	1.016
scale(bmi.diff)	0.878	0.785	0.978
scale(pqol.diff)	0.988	0.965	1.011
scale(mqol.diff)	1.014	0.994	1.035

Annotations: OR = odds ratio, 95%CI = 95% confidence interval, LL = lower limit, UL = upper limit

Note that all scaled variables have been standardized with mean equal to zero and standard deviation equal to one.
